# Supplementary material for: A Framework for Interfacing and Partnering with Environmental Justice Communities as a Prelude to Human Health and Hazard Identification in the Vulnerable Census Tracts of Columbus, Ohio
Source: Int J Environ Res Public Health. 2022 Oct 25;19(21):13846. doi: 10.3390/ijerph192113846 (PMC9654058; doi:10.3390/ijerph192113846)
Supplement: Supplementary file 1 [file ijerph-19-13846-s001.zip › ijerph-1965053-supplementary.pdf]

Environmental Exposure History Questionnaire
Division of Environmental Health Sciences

COMMUNITY

For each of the items listed below:	Do you presently live nearby	If you ever lived nearby, please write the years.
1. Heavy traffic	□ No □ Yes *(please specify)* ○ highway ○ busy street	____________
2. Vehicle idling area	□ No □ Yes *(please specify)* ○ auto ○ bus/truck	____________
3. Dump site	□ NO □ Yes *(please specify type)* ____________	____________
4. Farm(s)	□ No □ Yes *(please specify type)* ____________	____________
5. Industrial plant(s)	□ No □ Yes *(please specify type)* ____________	____________
6. Polluted lake/stream	□ No □ Yes *(please specify type)* ____________	____________
7. Nuclear power plant	□ No □ Yes	
8. Hydro towers	□ No □ Yes	

9. Other potential hazards □ No □ Yes *(please specify type)* ______________ ________

10. Do you protect yourself from excess sun exposure? □ rarely □ occasionally □ often □ always

HOME & HOBBY

11. **How long have you lived in your present residence?** ____________**How old is it?** __________

12. **What type of dwelling is your residence?** □ house □ mobile home
□ apartment → ○ basement ○ 2nd floor ○ high rise → floor

13**. Ownership?** □ owner occupied □ rental □ public housing

14**. Do you use:** □ central vacuum? □ HEPA filter vacuum? □ other vacuum? ____________

15**. Have you done any renovating?** □ No □ Yes → When? ______________________

What? ______________________

16**. Do you own/lease a car?** □ No □ Yes → Age? _____ **Smoking permitted inside?** ○ No ○ Yes

17. **Do you use pesticide or herbicides (bug or weed killer, flea/tick sprays, collars, powders, pellets, etc.):**

A in your home? □ No □ Yes *(please specify type)* ______________________
B on your pets? □ No □ Yes *(please specify type)* ______________________
C on your lawn or garden? □ No □ Yes *(please specify type)* ______________________

SCHOOL (if applicable)

18. How old is your or your child's school? _________ Number of floors: _______Number of occupants: _________

19. Have additions been made to the original building? ◻ No ◻ Yes → When? ___________________________

20. Number of portable classrooms in use: _______________

21. Hours per day you or your child spends in a portable classroom: _______________

22. School neighborhood: ◻ rural ◻ suburban ◻ urban

23. Is your or your child's school located near any of the following:

Heavy traffic ◻ No ◻ Yes *(please specify)* ○ highway ○ busy street

Vehicle idling area ◻ No ◻ Yes *(please specify)* ○ auto ○ bus/truck

Dump site ◻ No ◻ Yes *(please specify type)* ____

Farm(s) ◻ No ◻ Yes *(please specify type)* ____

Industrial plant(s) ◻ No ◻ Yes *(please specify type)* ____

Polluted lake/stream ◻ No ◻ Yes *(please specify type)* ____

Nuclear power plant ◻ No ◻ Yes

Hydro towers ◻ No ◻ Yes

Other potential hazards ◻ No ◻ Yes *(please specify type)* ____

24. **Which of the following does you or your child's school have?** (Please check all that apply*)*

◻ carpeted classrooms ◻ central air conditioning ◻ art room-exhaust hood? ○ No ○ Yes
◻ unvented copy machine(s) ◻ windows that open ◻ laboratory-exhaust hood? ○ No ○ Yes
◻ flaking paints ◻ moldy smell ◻ workshop-exhaust hood? ○ No ○ Yes

25. **Have any of the following occurred in your or your child's school during the current or last school year?** *(Please check all that apply)*

◻ carpet cleaning ◻ construction ◻ renovation ○ painting
◻ new flooring or furniture *(please specify)* _______________ ◻ flood, water leaks
◻ roof tarring ◻ use of pesticides / herbicides → ○ indoors ○ outdoors

26. **Are the following products used in your or your child's school during the school year?** *(Please check all that apply)*

◻ deodorizer strips ◻ furniture wax or polish ◻ odorous cleaning products
◻ floor wax ◻ scented washroom soap ◻ spray paints
◻ permanent markers ◻ strong-smelling art supplies

27. **Does you or your child's school have a policy regarding the use of personal scented products by staff and students?**

□ No □ Yes *(please specify)* → ○ prohibition of scented products ○ encouragement of unscented products

Occupational Exposure Inventory

(Please check all that apply)

28. Have you ever been off work for more than 1 day because of an illness related to work? □ No □ Yes

29. Have you ever been advised to change jobs or work assignments because of any health problems or injuries? □ No □ Yes

30. Has your work routine changed recently? □ No □ Yes

31. Is there poor ventilation in your workplace? □ No □ Yes

Environmental History

(Please check all that apply)

32. Which of the following do you have in your home?

□ Air conditioner □ Air purifier □ Central heating (gas or oil?)

□ Fireplace □ Wood stove □ Humidifier

33. Have you recently acquired: new furniture or carpet □ No □ Yes

Refinished furniture □ No □ Yes Remodeled your home □ No □ Yes

34. Have you weatherized your home recently? □ No □ Yes

35. Do you (or any household member) have a hobby or craft? □ No □ Yes

36. Do you work on your car? □ No □ Yes

37. Have you ever changed your residence because of a health problem? □ No □ Yes

38. Does your drinking water come from a private well, □ No □ Yes,
city water supply □ No □ Yes or grocery store □ No □ Yes

If you answered *yes* to any of the questions, please explain below.

39. **For each of the items listed below:** **Do you presently have in your <u>HOME</u>?**

Pets □ No □ Yes *(please specify kind & number)* _____ ______

Pets sleep in your bedroom □ No □ Yes

Indoor plants □ No □ Yes → *How many?* ______ ______

40. **Do you use dust mite-proof: Pillow cover(s)?** □ No□ Yes **Mattress cover(s)?** □ No □ Yes

41. **What hobbies do you have?** ____

____

____

42. **What hobbies do members of your household have?** ____

____

____

43. **Have you ever personally done any of the following:**

□ furniture stripping/refinishing □ No □ Yes Years: _________

□ home renovating □ No □ Yes Years: _________ *(please specify type)*

□ art work (*e.g. painting, ceramic stained glass, leather work, etc.)* □ No □ Yes Years: _________ *(please specify type)*

□ other non-occupational activities with exposure to toxic chemicals □ No □ Yes Years: ________ *(please specify type)*

PERSONAL

Natural Inhalant Allergies

44. **Do you think you are allergic to any seasonal pollens, animal danders, dust, mites, or molds?**

□ No □ Yes *(please specify which)* ____

45. **Have you ever had allergy tests?** □ No □ Yes

If **YES,** please specify:

Age	Year	Type of Test	Results	Treatments (e.g. avoidance, shots, Medications)	Improvement 0=worse 1=none 2=a little 3=some 4=a lot

Synthetic Chemicals

46. **Have you ever had symptoms you linked with exposure to any synthetic (man-made) chemicals at a level that did not seem to bother most people (e.g. paints, perfumes, cosmetics, diesel exhaust, jet fuel, tar, etc.)?**

□ No □ Yes

'Linked' means that the symptom started or worsened within 48 hours after you were exposed to something, or the symptom improved or disappeared after you were no longer exposed to it.
'Exposure' means being near, touching, smelling, breathing in, eating, drinking, swallowing or injecting something.

If **YES**, please specify chemical(s) and symptom(s):

Man-made Chemical	Symptoms Linked with Low Level Exposure	Presently Affected? 1= a little 2=somewhat 3=a lot	In the Past 1=a little 2=somewhat 3=a lot

47. **How often do you use SCENTED personal products?** *(please check)*

**Scented Products**	Soap	Lotion	Cosmetics	Hair permanent	Hair tint	Perfume/aftershave	Other(s) *(please specify)*
Never	□	□	□	□	□	□	□ ____________
Occationally	□	□	□	□	□	□	□ ____________
Daily	□	□	□	□	□	□	□ ____________

Artificial Materials

48. **How many metal dental fillings / caps do you currently have?** silver / mercury ________gold _________

49. **Have you had silver / mercury fillings removed?** □ No □ Yes → Number removed: ____ Year(s): ____

50. **Do you have other artificial materials in your body (e.g. pins, screws, plates, meshes, valves, implants, etc.)?**

□ No □ Yes *(please specify)* ____

Smoking History

51. **Do you currently use tobacco (daily or almost every day)?**

□ No □ Yes *(please specify)* → ○ cigarettes ○ cigars ○ pipe ○ snuff ○ chewing tobacco

- If **Yes** average number per day: __________________ Average number per day: ____________________
- If **No** , have you ever used tobacco (daily or almost every day)? ○ No ○ Yes
  If **YES**, number of years you used tobacco: _______________ Average number per day: ____________
  Date you last used tobacco regularly: Year _______________

Demographics

52. How old are you?
----18-29
----30-39
----40-49
----50-59

53. What is your highest level of education?
----Did not graduate from high school
----Graduated high school
----Some college
----Graduated college
----Some postgraduate education
----Professional degree

54. **What is your zip code?** ____________
